# Correction: Ishikawa et al. Systematic Review of Beta-Lactam vs. Beta-Lactam plus Aminoglycoside Combination Therapy in Neutropenic Cancer Patients. *Cancers* 2024, *16*, 1934

**DOI:** 10.3390/cancers16122274

**Published:** 2024-06-19

**Authors:** Kazuhiro Ishikawa, Tomoaki Nakamura, Fujimi Kawai, Erika Ota, Nobuyoshi Mori

**Affiliations:** 1 Department of Infectious Diseases, St. Luke’s International Hospital, Tokyo 104-8560, Japan; morinob@luke.ac.jp; 2 Department of Pulmonary Medicine, Thoracic Center, St. Luke’s International Hospital, Tokyo 104-8560, Japan; tonaka@luke.ac.jp; 3 Library, Department of Academic Resources, St. Luke’s International University, Tokyo 104-0044, Japan; 4 Global Health Nursing, Graduate School of Nursing Sciences, St. Luke’s International University, Tokyo 104-0044, Japan; otae@luke.ac.jp; 5 Tokyo Foundation for Policy Research, Tokyo 106-0032, Japan

## Text Correction

There was an error in the original publication. The authors wish to revise one sentence on Abstract, which was overlooked in the final proofreading.

A correction has been made to Abstract: Regarding treatment failure, combination therapy showed no significant differences compared with monotherapy (RR 0.99, 95% CI 0.94 to 1.03, moderate certainty of evidence).

## Error in Table

In the original publication [1], there was a mistake in Table 2 as published. Table 2 should include relevant data on all-cause mortality, infection-related mortality, treatment mortality, and treatment failure. The corrected Table 2 appears below. The authors state that the scientific conclusions are unaffected. This correction was approved by the Academic Editor. The original publication has also been updated.

## Figures and Tables

**Table 2:** Summary of findings regarding the administration of beta-lactam compared to beta-lactam plus aminoglycoside for febrile neutropenia.

Beta-Lactam Compared to Beta-Lactam + Aminoglycoside for Febrile Neutropenia						
Patient or population: febrile neutropenia; Intervention: beta-lactam; Comparison: beta-lactam + aminoglycoside						
Outcomes	Anticipated absolute effects * (95% CI)		Relative effect (95% CI)	№ of participants (studies)	Certainty of the evidence (GRADE)	Comments
	Risk with beta-lactam + aminoglycoside	Risk with beta-lactam				
All-cause mortality	81 per 1000	80 per 1000 (68 to 94)	OR 0.99 (0.84 to 1.16)	7023 (38 RCTs)	⨁⨁⨁⨁ High	
Infection-related mortality	50 per 1000	42 per 1000 (33 to 53)	OR 0.83 (0.66 to 1.05)	6469 (34 RCTs)	⨁⨁⨁⨁ High	
Treatment failure	420 per 1000	416 per 1000 (395 to 432)	OR 0.99 (0.94 to 1.03)	9285 (53 RCTs)	⨁⨁◯◯ Moderate ^a,b^	
Ag any dosing regimen	54 per 1000	25 per 1000 (19 to 32)	RR 0.46 (0.36 to 0.60)	6007 (28 RCTs)	⨁⨁⨁⨁ High	

* The risk in the intervention group (and its 95% confidence interval) is based on the assumed risk in the comparison group and the relative effect of the intervention (and its 95% CI). CI: confidence interval; OR: odds ratio; RR: risk ratio; Ag: aminoglycoside; a. Differences decreased with low risk of bias regarding allocation concealment. b. Differences in effects between published and unpublished trials. GRADE Working Group grades of evidence. High certainty: we are very confident that the true effect lies close to the estimated effect. Moderate certainty: we are moderately confident in the estimated effect: the true effect is likely to be close to the estimated effect, but there is a possibility that it is substantially different. Low certainty: our confidence in the estimated effect is limited: the true effect may be substantially different from the estimated effect. Very low certainty: we have very little confidence in the estimated effect: the true effect is likely to be substantially different from the estimated effect.
