# Supplementary material for: Systematic Review of Beta-Lactam vs. Beta-Lactam plus Aminoglycoside Combination Therapy in Neutropenic Cancer Patients
Source: Cancers (Basel). 2024 May 19;16(10):1934. doi: 10.3390/cancers16101934 (PMC11487387; doi:10.3390/cancers16101934)
Supplement: Supplementary file 1 [file cancers-16-01934-s001.zip › cancers-3021335-supplementary.pdf]

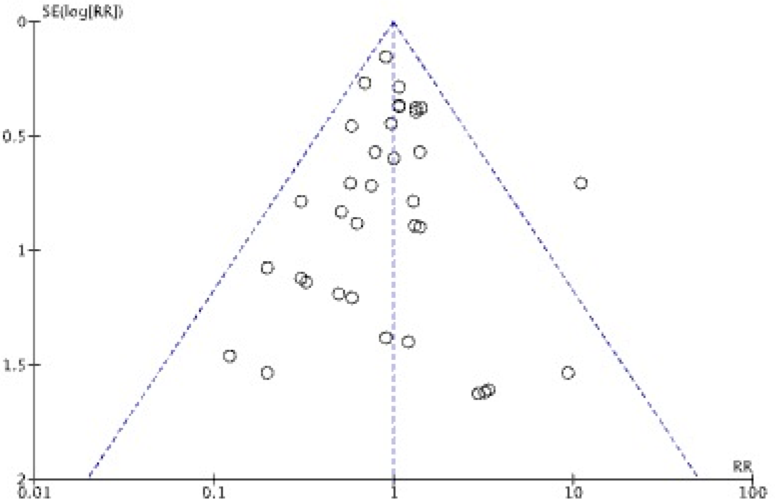

Figure S1. a The funnel plot of all-cause mortality in all beta-lactam.

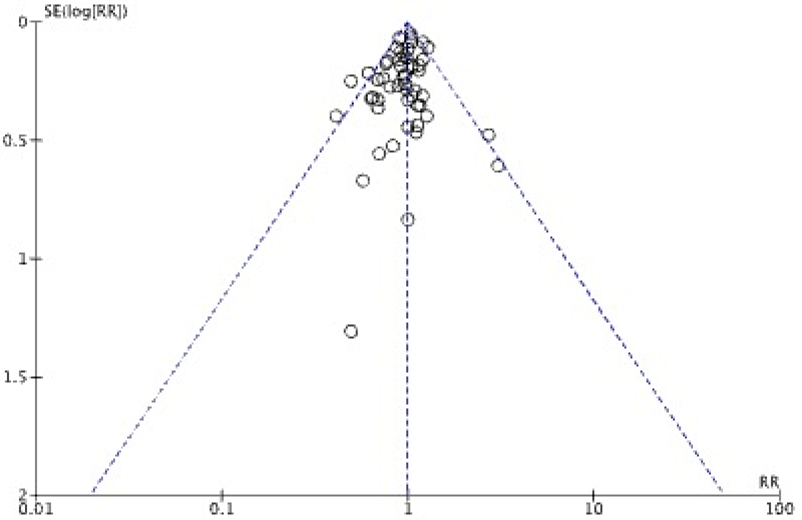

Figure S1. b The funnel plot of treatment failure in all beta-lactam.

Table S1. Characteristics of the included randomized controlled trials**.**

	Articles	Published country	Patient characteristics	Intervention	Comparison	definition of treatment failure or success	description of ratio of GNB resistant strain
1	Agaoglu 2001 [1]	Turkey	children/leukemia(85.1%), solid tumor(14.9%)	MEPM	CFPM+ netilmicin/ CAZ+ AMK	success: fever and clinical signs of infection(whether present) resolved and the infecting organisms(whether isolated) were eradicated without change of the antibiotics	MRSA(13%), Not reported to the resistant of GNB
2	Akova 1999[2]	Turkey	adult/ leukemia(61%), lymphoma(12%), solid tumor(22%) excluding allogeneic BMT	MEPM	CAZ+AMK	the addition of any antibacterial agent to the initial regimen because of persistent fever or in case of death due to infection.	In blood, MRSE(2 vs 1). In urine, MRSE(1 vs 0), MRSA(1 vs 0), Not reported to the resistant of GNB (unit: strain)
3	Anoshirvani 2018 [3]	Iran	adult/ leukemia(41%), lymphoma(20%), solid tumor(31%)	IMP	CAZ+AMK	clinical response: resolution of fever within 72 hours	susceptibility IMP/CS 100%, CAZ 100%, AMK 86.6 %

4	Antmen 2001 [4]	Turkey	adult/ leukemia(77),lymphoma(6), neuroblastoma(17)	MEPM	CAZ+AMK	overall success rate without treatment modification(information only from abstract[4])	NR
5	Behre 1998[5]	Germany	adult/ leukemia(30%), lymphoma(22%), solid tumor(45%)	MEPM	CAZ+AMK	Treatment failure: cured with treatment modification, unchanged/worse in the end of study therapy, and relapse during follow-up	excluding proven resistant to MEPM or CAZ/AMK in the study,/two resistant-strains to MEPM, one resistant strain to CAZ+AMK
6	Cometta 1996 [6]	Europe	adult, children/leukemia(59%),lymphoma(18%), solid tumor(16%)	MEPM	CAZ+AMK	(i) the patient died of the primary infection, (ii) bacteremia persisted beyond the first 24 h of therapy, (iii) breakthrough bacteremia was documented, (iv) the documented pathogen was resistant to the allocated beta-lactam regardless of the evolution of the patient's clinical condition, or (v) no response was seen after at least 72 h of empiric therapy, and so on. Follow-up period: 30 days.	resistant to the study drugs<br>CNS (34 VS 33)<br>Enterococcus spp.(3 vs 2)<br>S. aureus (1 VS 4)<br>Corynebacterium spp. (2 VS 1)<br>Clostridium spp. (1 VS 1)<br>Streptococcus spp. (2 VS 0)<br>Corynebacterium jeikeium(0 VS 1)<br>Stomatococcus spp. (0 VS 1)<br>S. maltophilia (1 vs 1)<br>Enterobacter spp. (1 VS 1)<br>Acinetobacter spp. (1 VS 1)<br>(unit: strain)
7	Conte 1996 [7]	Chile	adult/ leuekimia(88%)	CPZ/SBT	CAZ+AMK	NR	NR
8	Corapcioglu 2005 [8]	Turkey	children/leukemia(64%), solid tumor(26%)	CFPM	CAZ+AMK	NR	all gram-negative isolates susceptible to CFPM, CAZ/AMK
9	Cornely 2001 [9] [10]	Germany	adult/ leukemia(64%), lymphoma(36%)	CFPM	PIPC/TAZ+AG	treatment modification at 72 hour	NA
10	De la Camara 1997 [11]	Spain	adult/hematological malignancy, leukemia (43%), BMT (49%)	MEPM	CAZ+AMK	unchanged/worse (no improvement or deterioration of signs and symptoms of the infection) and cure with modification (complete remission of local and systemic signs and symptoms of infection following the addition of another antibacterial). If any additional antibiotic was given before 72h, the response was always classified as unchanged/worse. The addition of an antibacterial or antifungal agent was considered as failure if the patient had an unexplained fever. Deaths due to infection were classified as treatment failures unless the patient received only one or two doses of the study	susceptibility to MEPM, CAZ, AMK : 50%, 38.7%, 45.1% for all Gram-positive bacteria; 44%, 36% and 52% for coagulase-negative staphylococci; and 90%, 90% and 70% for Gram-negative bacteria.

						antibiotic. Follow-up period: 1 month following end of treatment.	
11	De Pauw 1994[12] [13]	Australia, Canada, Europe	adult/ leukemia(75%), BMT(15%)	CAZ	PIPC+TOB	treatment modification + death due to infection	GNR CAZ: E. cloacae, PIPC/TOB:E. coli, K. pneumoniae
12	Del Favero 2001 [14]	Italy	adult/ leukemia(62%), lymphoma(19%), solid tumor(8%), , BMT(27%)	PIP/TAZ	PIPC/TAZ+AMK	persistent or relapsing fever, resistant pathogen, deterioration of clinical conditions, breakthrough bacteremia, withdrawal of therapy as a result of toxicity	susceptibility PIP/TAZ VS PIPC/TAZ+AMK Staphylococci(32% vs 30%), Streptococci, Enterococci(83% vs 100%), Other GPC(100% vs 0%), E. coli(85% vs 79%), Other Enterobacteriaceae(78% vs 71%), Pseudomonas spp.(50% vs 44%), Other GNB (50% vs 80%), AMK Pseudomonas spp.(50% vs 58%), Other GNB(95% vs 83%)
13	Dincol 1998 [15]	Turkey	adult/ lymphoma(43%), solid tumor(57%), excluded >7 days neutropenia and/or documented microbiological infection	IMP/CS	CPZ/SBT+AMK	treatment modification	Susceptibility rate gram-positive bacteremia: IMP/CS96%, CPZ/SBT 88%, AMK 78% GNB: IMP/CS 95%, CPZ/SBT 90%, AMK 81%
14	Doyen 1983 [16]	Belgium	adult/hematological malignancy	CAZ	CAZ+AMK	Body temperature drops 2 degrees Celsius or below 37.5 degrees Celsius within 72 hours of treatment	NR
15	Duzova 2001 [17]	Turkey	children/ lymphoma (63%), solid tumor(37%)	MEPM	PIPC+AMK	treatment modification	MRSA 2 strains, GNB: Not reported
16	El Haddad 1995 [18]	Egypt	children/ leukemia(63%), lymphoma(37%)	CPZ/SBT	PIPC+AMK	treatment success: defervescence with treatment	NR
17	Esteve 1997 [19]	Spain	adult/ hematological malignancy(excluding BMT)	P/T	PIPC/TAZ+AMK	NR	NR
18	Gaytan-Martinez 2002 [20]	Mexico	adult/non-Hodgkin lymphoma or acute leukemia	CFPM	CAZ+AMK	NR	NR
19	Gibson 1989 [21]	Australia	adult/ leukemia(60%), lymphoma(20%)	CAZ	azlocillin +AMK	NR	CAZ group-Serratia marcescens(throat, resistant to CAZ, AMK. azlocillin), combination group-P. aeruginosa (Hickman's catheter, resistant to AMK, azlocillin), S. epidermiditis (urine, AMK,), E. cloacae(throat, resistant to azlocillin, CAZ), excluding pathogens known to be resistant to any of the trial drugs
20	Gribble 1983 [22]	Canada	adult/cancer patient including neutropenia	PIPC	carbenicillin+GM	treatment failure: no response to treatment, or relapse during treatment or the follow-up period	PIPC-11/26, Carbenicillin+GM(4/24) including non-neutropenic patients, Enterococcus

						(14 days or untill discharge)	(3 vs 0), S. aureus(1 vs 0), S. epidermiditis(2 vs 3), E. coli(3 vs 0), Klebsiella(3 vs 0), Pseudomonas(3 vs 0), Citrobacter(1 vs 0), Enterobacter(1 vs 0), Candida(1 vs 1), excluding the pathogen resistant to study drugs in the study
21	Hense 2000 [23] [24]	Germany	adult/hematological malignancy	MEPM	CAZ+AMK	NR	NR
22	Hess 1998 [25]	Switzerland	adult/ leukemia(45%), lymphoma(22%), solid tumor(31%), ASCT(19%)	PIPC/TAZ	CAZ+AMK	success: if fever and clinical signs of infections resolved and if blood or infection sites were cleared from isolated pathogens without any change in the study treatment	excluding if resistant to PIPC/TAZ, CAZ/AMK in the study, MRSE(6 vs 5), Not reported to GNB
23	Hung 2003 [26]	Taiwan	children/cancer	MEPM	CAZ+AMK	Antimicrobial modification + initial success with further infection	susceptibility CAZ/AMK 100%, MEPM 100%
24	Jacobs 1993 [27]	USA	children/cancer	CAZ	CAZ+TOB	no apparent response to therapy in the absence of another definitive etiology not amenable to the study drugs (at the end of therapy)	4 case (CAZ: P. fluoorescens, S. aureus, CAZ/TOB: S.mitis, MRSE, S.aureus), excluding the pretreatment pathogen resistant to study drugs in the study
25	Jimeno 2006 [28] [29]	Spain	adult/solid tumor	CFPM	CAZ+AMK	success with treatment modification +failure(fever>3days, persistent bacteremia + death from sepsis)	all susceptible strain to the study drugs
26	Kamonrattana 2019 [30]	Thailand	children/cancer patient: leukemia(55%), lymphoma(7%),	PIPC/TAZ	CAZ+AMK	persistent fever more than 48 hours, recurrent infection within 1 week after discontinuation of antimicrobial therapy, modification of initial treatment protocol or death from infections	susceptibility MEPM 100%, CAZ 100%
27	Kiehl 2001 [31]	Germany	adult/ASCT	PIPC/TAZ	PIPC/TAZ+ netilmicin	NR	NR
28	Kinsey 1990 [32]	UK	adult, children/hematological malignancy	CAZ/CAZ+TEIC	CAZ+GM/CAZ+GM+TEIC	treatment modification	NR
29	Kliasova 2001 [33]	Russia	adult/hematological malignancy	MEPM	CAZ+AMK	NR	NR
30	Kojima 1994 [34]	Japan	adult/solid tumor	IMP/CS	IMP/CS+AMK	if there was no or minimal response to antimicrobial	NR
31	Leyland 1992 [35]	UK	adult/ leukemia(74%), lymphoma(21%)	IMP/CS	PIPC+GM	Treatment failure: temperature response, or were un-assessable, administrating of antimicrobial agents)including antifungal and antivirals)	IMP 1 (strain), PIPC 17, GM 9 PIPC/GM 5, but none of the patients with infections caused by study drugs, excluding the pretreatment pathogen resistant to study drugs in the study
32	Lieschke 1990 [36]	Australia	adult/cancer	IMP/CS	PIPC+TOB	NR	NR

33	Liu 1989 [37]	China	adult/leukemia(40%), lymphoma(27%), solid tumor(27%)	IMP/CS	CAZ+AMK	success with alternative antibiotic, failure of alternative treatment	NR
34	Madasamy 2016 [38]	India	adult, children/hematological, solid tumor,	CFPM	SBT/CPZ+AMK	treatment success: defervescence within 72 hrs, afebrile period continued for more than 48 hrs, no requirement for 2nd line antimicrobials	NR
35	Marie 1991 [39] [40] [41]	France	adult/ 2/3 chemotherapy for leukemia 1/3 conditioning regime alone or with TBI followed by BMT for leukemia, lymphoma or solid tumor	CAZ	CAZ+AMK	NR	CAZ+AMK: CAZ resistant in 7 gram-negative strains, 11 gram-positive strains
36	Miller 1993 [42]	USA	adult/ leukemia/lymphoma(47%), solid tumor(53%)	IMP/CS	CAZ+TOB	success: without modification	NR
37	Morgan 1983 [43]	England	children/leukemia(68%), solid tumor(26%)	CAZ	azlocillin +TOB	treatment failure: persistence of fever for more than five days with or without clinical deterioration + not evaluable + fatal + additional antimicrobial treatment	CAZ Resistant-P. aeruginosa,<br>TOB Resistant-S. epidermiditis, Viridians streptococcus, S. pneumoniae Azocillin Resistant-4 S. aureus, S. epidermiditis, Klebsiella spp.
38	Norrby 1987 [44] [45]	Europe Canada"	adult/ leukemia(67%), lymphoma(15%), solid tumor(6%)	IMP/CS	PIPC+AMK	NR	2 P. aeruginosa to AMK, CAZ
39	Novakova 1990 [46]	Netherlands	adult/leukemia(72%), lymphoma(10%), solid tumor(12%)	CAZ	PIPC+AMK	success: no modification	one Viridians streptococci, 2 CNS resistant to PIPC
40	Novakova 1991 [47]	Netherlands	adult/ leukemia(70%)	CAZ	CAZ+AMK	failure(the patient died due to the infection, and all signs and symptoms did not disappeared with or without any change of initial therapy)+response after therapy modification(the patient survived the infection, but defervescence and resolution of all signs and symptoms of infection occurred only after modification of the empiric regimen)	NR
41	Ozyilkan 1999 [48]	Turkey	adult/ leukemia(80%), lymphoma(10%), solid tumor(7%)	IMP/CS	CPZ/SBT+AMK	treatment modification at 72 hour	NR
42	Pacheco-Rosas, 2019 [49]	Mexico	children/hematological malignancy	PIPC/TAZ	PIPC/TAZ+AMK	persistence of fever and/or clinical evidence of infection on the fourth day after initiation of treatment, or death attributed to infection.	NR
43	Papachristodoulou 1996 [50]	Greece	adult/cancer	CAZ	CAZ+AMK	7 days persistent fever with neutrophil count>1,000	NR
44	Perez 1995 [51]	Chile	adult/ leukemia(73%), lymphoma(8%), solid tumor(12%)	IMP/CS	CAZ+AMK	treatment modification	NR

45	Piccart 1984 [52]	Belgium	adult/ hematological malignancy(55%), solid tumor(45%),	CPZ	CPZ+AMK	Death during therapy and clinical deterioration requiring a change in antimicrobial treatment	resistant CAZ group: one P .aeruginosa, CAZ+AMK group: two P. aeruginosa
46	Ponraj 2018[53]	India	adult, children/ leukemia(68%), lymphoma(16%)or solid malignancy(17%) excluding induction therapy of AML and SCT	CFPM	CPZ/SBT+AMK	not meet all the following criteria: afebrile within 72 h of starting antimicrobials, persistent afebrile status more than 48 h and no requirement of second-line antimicrobials and antifungal agents	MDR GNB 51% of total MDI
47	Rolston 1992 [54]	USA	adult/ hematological malignancy, mainly AML(66%), solid tumor(34%)	CAZ or IMP/CS	CAZ or IMP/CS + AMK	if the patient's infection showed no response or worsened while the patient was receiving the initial regimen and requiring a change in antibiotic therapy	one S. maltophilia resistant to CAZ two P. aeruginosa resistant to AMK
48	Schuchter 1988 [55]	USA	adult. children/BMT	CAZ	Ticarcillin+GM	treatment success: completely treated with empirical therapy	NR
49	Tamura 2002 [56]	Japan	adult/ leukemia(69%), lymphma(18%),	CFPM	CFPM+(AMK or Isepamicin or TOB or Netilmicin)	no response when no improvement in fever or clinical symptoms was observed at day 7	NR
50	Tamura 2004 [57]	Japan	adult/leukemia(48%), lymphoma(38%)	CFPM	CFPM+AMK	bacteremia, clinically diagnosed infection, fever of unknown origin	excluding infection with pathogen resistant to CFPM
51	Wade 1987 [58] [59] [60]	USA	adult/cancer	IMP/CS	PIPC+AMK	NR	NR
52	Wrzesien-Kus 2001 [61]	Poland	adult/95% hematologic malignancy	CFPM	CFPM+AMK	NR	NR
53	Yamamura 1997 [62] [63]	USA	adult/leukemia(29%), lymphoma(28%) excluding aplastic anemia, CML in blast crisis	CFPM	PIPC+GM	Treatment modification	NR
54	Yildirim 2008 [64]	Turkey	children/ ALL(87%), AML(21%),	IMP/CS or MEPM	PIPC/TAZ+AMK	treatment modification	Susceptibility PIPC/TAZ 15/20, Carbapenem 14/20, AMK 6/20
55	Zengin 2011 [65]	Turkey	children/ ALL(83%), AML(17%),	PIPC/TAZ	PIPC/TAZ+AMK	Treatment modification	PIPC/TAZ one GNB, three ESBL (K. pneumoniae, E. cloacae)

Abbreviations: AML, acute myelogenous leukemia; CAZ, ceftazidime; CFPM, cefepime; MEPM, IMP/CS, imipenem/cilastatin; meropenem; PIPC/TAZ, piperacillin/tazobactam; CPZ/SBT, cefoperazone/sulbactam; AG, aminoglycoside; AMK, amikacin; TOB, tobramycin; GM, gentamycin; BMT, bone mallow transplantation; GPC, gram positive coccus; GNB, gram negative bacilli; MDR, multi drug resistance; MDI, microbiological documented infection; ESBL, Extended spectrum beta-lactamase;ALL, acute lymphoblastic leukemia; AML, acute myeloblastic leukemia; CML, chronic myeloblastic leukemia; E. coli, Escherichia coli; K. pneumoniae, Klebsiella pnueumoniae; E. cloacae, Enterobacter cloacae, GPC, gram positive coccus; ASCT, autologous stem cell transplantation; P. fluoorescens. Pseudomonas fluoorescens; S. aureus, Staphylococcus aureus; S.mitis, Streptococcus mitis; MRSE, Methicillin-resistant Staphylococcus epidermiditis; P. aeruginosa, Pseudomonas aeruginosa; V. streptococcus, Viridians streptococcus; S. pneumoniae, Streptococcus pneumoniae.

Table S2. The characteristics of Aminoglycoside in each.

Author	Published year	Dose of Aminoglycoside	result of duration of therapy in AG combined arm	definition of renal failure	monitoring serum AG level

once daily					
Cometta	1996	AMK 20 mg/kg	7 days	Cr 50% increasing or >=45 mmol/L(adults only))	yes
Esteve	1997	AMK 15mg/kg	4 days	Cr 50% increasing	not mentioned
Dincol	1998	AMK 15mg/kg	8.9 days	not mentioned	not mentioned
Hess	1998	AMK 15mg/kg	7.4 days	at least twice the upper limit of the normal range	yes
Ponraj	2018	AMK 15mg/kg	not mentioned	not mentioned	not mentioned
Pacheco-Rosas	2019	AMK 20 mg/kg	not mentioned	Cr>0.5mg/dL increasing	not mentioned
Multiple daily					
Doyen	1983	not mentioned	not mentioned	not mentioned	not mentioned
Norrby	1987	AMK 15mg/kg in two or three divided doses	documented infection 12±6, clinically evaluation w/o documented infection 10±5	Cr >=1.3mg/dL (baseline 0.8-1.3) or Cr =0.5mg/dL increasing (baseline>=1.5mg/dL)	yes
Liu	1989	AMK 15mg/kg every 12hr	9.2 days	Cr>0.5mg/dL increasing	not mentioned
Lieschke	1990	TOB 1mg/kg/dose every 8 hr	9.5 days	not mentioned	not mentioned
Marie	1991	AMK 7.5 mg/kg/dose every 12hr	not mentioned	not mentioned	not mentioned
Novakova	1991	AMK 500mg every 8hr	16.9 days	Cr 50% increasing	not mentioned
Leyland	1992	GM 80mg every 8hr	7 days	not mentioned	yes
Rolston	1992	AMK 800 mg/m2/day as a continuous infusion over 24 hr	8 days	Cr>0.4mg/dL increasing	not mentioned
Jacobs	1993	TOB 2.5 mg/kg/dose every 8 hr or 80 mg/m2/day in three divided doses	not mentioned	not mentioned	yes
Miller	1993	TOB 3-5mg/kg/day in two or three divide dose	8.3 days	not mentioned	yes
De Pauw	1994	TOB 1.7 to 2 mg/kg every 8 h	16.6 days	Cr 50% increasing	yes
Kojima	1994	AMK 400mg/m^2/day in two divided doses	5.0 days	not mentioned	not mentioned
El Haddad	1995	AMK 15mg/kg in three divided doses	11.2 days	not mentioned	not mentioned
Conte	1996	AMK 15mg/kg in two divided doses	not mentioned	not mentioned	not mentioned
De la Camara	1997	AMK 15mg/kg in two or three divided doses	not mentioned	not mentioned	yes
Yamamura	1997	GM 1.5 mg/kg every 8 h	not mentioned	Cr>=0.5mg/dL increasing	yes
Behre	1998	AMK 15mg/kg in two or three divided doses	6 days	not mentioned	yes
Agaoglu	2001	netilmicin 5 mg/kg/day in two or three divided doses, AMK 15 mg/kg/day in 2 divided doses	not mentioned	not mentioned	not mentioned
Tamura	2002	AMK 200 mg, q12hr, isepamicin 200 mg, q12hr, TOB 90 mg q12hr, or netilmicin 100 mg, q12hr	not mentioned	WHO criteria for cancer treatment	not mentioned
Hung	2003	AMK 5mg/kg/dose every 8 hr	not mentioned	Cr 50% increasing or =>1.5mg/dL	not mentioned
Tamura	2004	not mentioned	not mentioned	WHO criteria for cancer treatment	not mentioned
Jimendo	2006	AMK 500 mg every 12 h	not mentioned	not mentioned	yes

Abbreviation: RCT, Randomized Control Studies; AMK, amikacin; GM, gentamicin; TOB, tobramycin.

Table S3. Sensitivity analysis of all-cause mortality by publication year and age groups.

published year	1983-2019	2000-2019	2010-2019

same beta-lactam	10 RCTs<br>RR 0.74<br>(95%CI, 0.53-1.06) I^2 0%	4RCTs<br>RR 0.75<br>(95%CI, 0.48-1.16) $I^2$6%	not applicable
different beta-lactam	28 RCTs<br>RR 0.97<br>(95%CI, 0.81-1.16) $I^2$0%	11RCTs<br>RR 1.20<br>(95%CI, 0.83-1.74) I^2 0%	4RCTs<br>RR 1.11<br>(95%CI, 0.67-1.84) I^2 0%
all beta-lactam	38 RCTs<br>RR 0.99<br>(95%CI, 0.84-1.16) I^2 0%	15RCTs<br>RR 0.99<br>(95%CI, 0.74-1.31) $I^2$0%	not applicable
age groups	all ages	children	adults
same beta-lactam	not applicable	not applicable	not applicable
different beta-lactam	28 RCTs<br>RR 0.97<br>(95%CI, 0.81-1.16) I^2 0%	5RCTs<br>RR 0.74<br>(95%CI, 0.29-1.90) $I^2$27%	20RCTs<br>RR 0.95<br>(95%CI, 0.77-1.17) I^2 0%
all beta-lactam	not applicable	not applicable	not applicable

Abbreviation: RR, risk ratio; RCT, Randomized Control Studies; CI, Confidence interval; I^2 heterogenicity.

Table S4. sensitivity analysis of infection-related mortality by publication year and age groups.

**published year**	**1983-2019**	**2000-2019**	**2010-2019**
same beta-lactam	8 RCTs<br>RR 0.67<br>(95%CI, 0.42-1.07) I^2 0%	4 RCTs<br>RR 0.53<br>(95%CI, 0.28-1.00)<br>I^2 0%	not applicable
different beta-lactam	26 RCTs<br>RR 0.86<br>(95%CI, 0.67-1.11) I^2 0%	10 RCTs<br>RR 1.19<br>(95%CI, 0.69-2.06) I^2 0%	2 RCTs<br>RR 1.00<br>(95%CI, 0.43-2.34)
all beta-lactam	34RCTs<br>RR 0.83<br>(95%CI, 0.66-1.05) I^2 0%	14RCTs<br>RR 0.84<br>(95%CI, 0.55-1.29) I^2 0%	not applicable
age groups	all ages	children	adults
same beta-lactam	8 RCTs<br>RR 0.67<br>(95%CI, 0.42-1.07) $I^2$0%		7 RCTs<br>0.68(95%CI, 0.43-1.10) I2 0%
different beta-lactam	26 RCTs<br>RR 0.86<br>(95%CI, 0.67-1.11) I^2 0%	7 RCTs<br>RR 0.53<br>(95%CI, 0.14-2.03) I^2 0%	17 RCTs<br>RR 0.90<br>(95%CI, 0.68-1.20) $I^2$0%
all beta-lactam	not applicable	not applicable	not applicable

Abbreviation: RR, risk ratio; RCT, Randomized Control Studies; CI, Confidence interval; I^2 heterogenicity.

Table S5. Sensitivity analysis of treatment failure by publication year and age groups.

**published year**	**1983-2019**	**2000-2019**	**2010-2019**
same beta-lactam	16 RCTs<br>RR 1.11<br>(95%CI, 1.02-1.20)<br>I^2 13%	6 RCTs<br>RR 1.05<br>(95%CI, 0.93-1.18) I^2 0%	not applicable
different beta-lactam	37 RCTs<br>RR 0.94<br>(95%CI, 0.89-0.99)<br>I^2 0%	14 RCTs<br>RR 0.94<br>(95%CI, 0.85-1.03) I^2 0%	4 RCTs<br>RR 0.92<br>(95%CI, 0.78-1.07) I^2 0%
all beta-lactam	53 RCTs	20 RCTs	not applicable

	RR 0.99 (95%CI, 0.94-1.03) I^2 14%	RR 0.98 (95%CI, 0.91-1.06) I^2 0%	
age groups	all ages	children	adults
same beta-lactam	16 RCTs RR 1.11 (95%CI, 1.02-1.20) I^2 13%	not applicable	14 RCTs RR 1.10 (95%CI, 1.01-1.19) I^2 1%
different beta-lactam	37 RCTs RR 0.94 (95%CI, 0.89-0.99) I^2 0%	7 RCTs RR 0.77 (95%CI, 0.62-0.95) I^2 20%	27 RCTs RR 0.96 (95%CI, 0.90-1.03) I^2 0%
all beta-lactam	not applicable	not applicable	not applicable

Abbreviation: RR, risk ratio; RCT, Randomized Control Studies; CI, Confidence interval; I^2 heterogenicity.

Table S6 Sensitivity analysis of the Impact of Serum aminoglycoside monitoring on nephrotoxicity in any daily regimen.

**aminoglycoside regimen**	**all RCTs**	**only monitoring serum AG RCTs**
AG any daily regimen	28 RCTs RR 0.46 (95%CI, 0.36-0.60) I^2 0%	11 RCTs RR 0.46 (95%CI, 0.34-0.62) I^2 5%

Abbreviation: AG aminoglycoside; RR, risk ratio; RCT, Randomized Control Studies; CI, Confidence interval; I^2 heterogenicity.

Content S1: Search strategy

MEDLINE Ovid

1 exp Neoplasms/
2 Bone Marrow Transplantation/
3 (cancer* or tumor* or tumour* or neoplas* or malignan* or carcinoma* or adenocarcinoma* or leukemia* or leukaemia* or bone marrow transplant*).mp.
4 1or2or3
5 exp Agranulocytosis/
6 (agranulocytosis or neutropen* or neutropaen* or granulocytopen* or granulocytopaen* or granulopen* or granulopaen*).mp.
7 5or6
8 exp beta-Lactams/
9 exp Anti-Bacterial Agents/
10 (beta-lactam* or antibiotic* or antimicrob* or anti-microb* or antibacteria* or antibacteria*).mp.
11 8 or 9 or 10
12 exp Aminoglycosides/
13 (aminoglycoside* or gentamicin or gentamycin or amikacin or amikacyn or tobramicin or tobramycin or kanamicin or kanamycin or netilmicin or netilmycin).mp.
14 12 or 13
15 4 and 7 and 11 and 14
16 randomized controlled trial.pt.
17 controlled clinical trial.pt.
18 randomized.ab
19 placebo.ab.
20 drug therapy.fs.
21 randomly.ab.

22 trial.ab.
23 groups.ab.
24 16 or 17 or 18 or 19 or 20 or 21 or 22 or 23
25 15 and 24

Embase

('neoplasm'/exp OR 'bone marrow transplantation'/exp OR cancer* OR tumor* OR tumour* OR neoplas* OR malignan* OR carcinoma* OR adenocarcinoma* OR leukemia* OR leukaemia* OR 'bone marrow transplant*') AND ('antiinfective agent'/exp OR 'beta lactam*' OR antibiotic* OR antimicrob* OR 'anti microb*' OR antibacterial* OR 'anti bacteria*') AND ('aminoglycoside antibiotic agent'/exp OR aminoglycoside* OR gentamicin OR gentamycin OR amikacin OR amikacyn OR tobramicin OR tobramycin OR kanamicin OR kanamycin OR netilmicin OR netilmycin) AND ('crossover procedure'/exp OR 'double-blind procedure'/exp OR 'randomized controlled trial'/exp OR 'single-blind procedure'/exp OR random* OR factorial* OR ((crossover* OR cross) AND over*) OR 'cross over*' OR placebo* OR (double* NEXT/1 blind*) OR (singl* NEXT/1 blind*) OR assign* OR allocat* OR volunteer*)

CENTRAL

#1 MeSH descriptor Neoplasms explode all trees
#2 MeSH descriptor Bone Marrow Transplantation, this term only
#3
(cancer* or tumor* or tumour* or neoplas* or malignan* or carcinoma* or adenocarcinoma* or leukemia* or leukaemia* or bone
marrow transplant*)
#4 (#1 OR #2 OR #3)
#5 MeSH descriptor Agranulocytosis explode all trees
#6 (agranulocytosis or neutropen* or neutropaen* or granulocytopen* or granulocytopaen* or granulopen* or granulopaen*)
#7 (#5 OR #6)
#8 MeSH descriptor beta-Lactams explode all trees
#9 MeSH descriptor Anti-Bacterial Agents explode all trees
#10 beta-lactam* or antibiotic* or antimicrob* or anti-microb* or antibacterial* or antibacteria*
#11 (#8 OR #9 OR #10)
#12 MeSH descriptor Aminoglycosides explode all trees
#13 (aminoglycoside* or gentamicin or gentamycin or amikacin or amikacyn or tobramicin or tobramycin or kanamicin or kanamycin or netilmicin or netilmycin)
#14 (#12 OR #13)
#15 (#4 AND #7 AND #11 AND #14)

References

1. Agaoglu, L.; Devecioglu, O.; Anak, S.; Karakas, Z.; Yalman, N.; Biner, B.; Eryilmaz, E.; Goksan, B.; Unuvar, A.; Agirbasli, H.; et al. Cost-effectiveness of cefepime + netilmicin or ceftazidime + amikacin or meropenem monotherapy in febrile neutropenic children with malignancy in Turkey. *J Chemother* **2001**, *13*, 281-287, doi:10.1179/joc.2001.13.3.281.
2. Akova, M.; Akan, H.; Korten, V.; Biberoglu, K.; Hayran, M.; Unal, S.; Kars, A.; Kansu, E. Comparison of meropenem with amikacin plus ceftazidime in the empirical treatment of febrile neutropenia: a prospective randomised multicentre trial in patients without previous prophylactic antibiotics. Meropenem Study Group of Turkey. *Int J Antimicrob Agents* **1999**, *13*, 15-19, doi:10.1016/s0924-8579(99)00096-5.
3. Anoshirvani, A.A.; Zarinfar, N.; Rafiee, M.; Zamani, Z. Effect of Combination Therapy of Ceftazidime/Amikacin and Monotherapy with Imipenem on the Treatment of Fever and Neutropenia in Patients with Cancers. *Open Access Maced J Med Sci* **2018**, *6*, 1423-1430, doi:10.3889/oamjms.2018.310.

4. Antmen, B. Infectious cost of leisure (Keynote lecture)-P1087 nitial empiric antibiotic treatments in childhood febrile neutropenia: meropenem vs. ceftazidime plus amikacin combination. **2022**, doi:10.1111/j.1469-0691.2001.tb00001.x.
5. Behre, G.; Link, H.; Maschmeyer, G.; Meyer, P.; Paaz, U.; Wilhelm, M.; Hiddemann, W. Meropenem monotherapy versus combination therapy with ceftazidime and amikacin for empirical treatment of febrile neutropenic patients. *Ann Hematol* **1998**, *76*, 73-80, doi:10.1007/s002770050366.
6. Cometta, A.; Calandra, T.; Gaya, H.; Zinner, S.H.; de Bock, R.; Del Favero, A.; Bucaneve, G.; Crokaert, F.; Kern, W.V.; Klastersky, J.; et al. Monotherapy with meropenem versus combination therapy with ceftazidime plus amikacin as empiric therapy for fever in granulocytopenic patients with cancer. The International Antimicrobial Therapy Cooperative Group of the European Organization for Research and Treatment of Cancer and the Gruppo Italiano Malattie Ematologiche Maligne dell'Adulto Infection Program. *Antimicrob Agents Chemother* **1996**, *40*, 1108-1115, doi:10.1128/AAC.40.5.1108.
7. Conte G, F.C.A.J.A.D.T.L.B.O.; et al. Single agent sulperazone vs. two agent ceftazidime-amikacin in high risk febrile neutropenic patients. *Blood* **1996**, *88(10):30b.*
8. Corapcioglu, F.; Sarper, N. Cefepime versus ceftazidime + amikacin as empirical therapy for febrile neutropenia in children with cancer: a prospective randomized trial of the treatment efficacy and cost. *Pediatr Hematol Oncol* **2005**, *22*, 59-70, doi:10.1080/08880010590896297.
9. Cornely Oa, R.D.B.D.M.G.W.M.C.X. Three-armed multicenter randomized study on the empiric treatment of neutropenic fever in a high risk patient population (PEG study III). *Proceedings of the 41st Interscience Conference on Antimicrobial Agents and Chemotherapy;* **2001**, *Abstract L-775. 2001.*
10. G, M. Cefepime in the empirical initial treatment of febrile neutropenic cancer patients [Cefepim in der empirischen Initial therapie bei febrilen neutropenischen Patienten mit mignen erkankungen]. *Chemotherapie Journal* **2004**, *13:174–180.*
11. de la Camara, R.; Figuera, A.; Sureda, A.; Hermida, G.; Verge, G.; Olalla, I.; Fernandez Ranada, J.M.; Domingo Albos, A. Meropenem versus ceftazidime plus amikacin in the treatment of febrile episodes in neutropenic patients: a randomized study. *Haematologica* **1997**, *82*, 668-675.
12. de Pauw, B.E.; Kauw, F.; Muytjens, H.; Williams, K.J.; Bothof, T. Randomized study of ceftazidime versus gentamicin plus cefotaxime for infections in severe granulocytopenic patients. *J Antimicrob Chemother* **1983**, *12 Suppl A*, 93-99, doi:10.1093/jac/12.suppl_a.93.
13. T, D.W. Antibiotic treatment in patients with haematological malignancies and bone marrow transplantation. *Perspectives on Therapeutics in Northern Europe.* **1986**, *46, London: Glaxo, 26 September:6–7.*
14. Del Favero, A.; Menichetti, F.; Martino, P.; Bucaneve, G.; Micozzi, A.; Gentile, G.; Furno, P.; Russo, D.; D'Antonio, D.; Ricci, P.; et al. A multicenter, double-blind, placebo-controlled trial comparing piperacillin-tazobactam with and without amikacin as empiric therapy for febrile neutropenia. *Clin Infect Dis* **2001**, *33*, 1295-1301, doi:10.1086/322646.
15. Dincol D, A.A.A.F.S.M.W.A.A.H.; et al. A comparison of imipenem monotherapy versus cefoperazone/sulbactam plus amikacin combination treatment in febrile neutropenic cancer patients. *Cancer Journal;11(2):89–93.* **1998**.
16. Doyen C, T.J.M.W.G.M.J.L. A randomized therapeutic trial for ceftazidime versus ceftazidime and amikacin in febrile granulopenic patients. *Proceedings of the 13th International Congress of Chemotherapy. Vienna: Spitzy KH, :26–29.* **1983**.
17. Duzova, A.; Kutluk, T.; Kanra, G.; Buyukpamukcu, M.; Akyuz, C.; Secmeer, G.; Ceyhan, M. Monotherapy with meropenem versus combination therapy with piperacillin plus amikacin as empiric therapy for neutropenic fever in children with lymphoma and solid tumors. *Turk J Pediatr* **2001**, *43*, 105-109.
18. Ama, E.H. Comparison of cefoperazone-sulbactam versus piperacillin plus amikacin as empiric therapy in pediatric febrile neutropenic cancer patients. *Current Therapeutic Research Clinical and Experimental ;56 (10):1094–9.* **1995**.
19. Esteve J, N.B.M.J.G.R.M.F.M.E. Piperacillin/tazobactam vs. piperacillin/ tazobactam plus amikacin as empiric therapy for fever in neutropenic patients. *Blood ;10(Suppl 1 (Pt 2)):229b. Abstract 3767.* **1997**.
20. Gaytan-Martinez Je, M.-G.E.C.L.J.F.-A.J.L.S.-C.E.M.-T.B.; et al. Efficacy of empirical therapy with cefepime compared with ceftazidime plus amikacin in febrile neutropenic patients. *Proceedings of the Annual Meeting of the American Society of Hematology. :Abstract No. 3655.* **2002**.
21. Gibson, J.; Date, L.; Joshua, D.E.; Young, G.A.; Wilson, A.; Benn, R.; Benson, W.; Iland, H.; Vincent, P.C.; Kronenberg, H. A randomised trial of empirical antibiotic therapy in febrile neutropenic patients with hematological disorders: ceftazidime versus azlocillin plus amikacin. *Aust N Z J Med* **1989**, *19*, 417-425, doi:10.1111/j.1445-5994.1989.tb00296.x.
22. Gribble, M.J.; Chow, A.W.; Naiman, S.C.; Smith, J.A.; Bowie, W.R.; Sacks, S.L.; Grossman, L.; Buskard, N.; Growe, G.H.; Plenderleith, L.H. Prospective randomized trial of piperacillin monotherapy versus carboxypenicillin-aminoglycoside combination regimens in the empirical treatment of serious bacterial infections. *Antimicrob Agents Chemother* **1983**, *24*, 388-393, doi:10.1128/AAC.24.3.388.
23. Hense J, B.H.L.J.M.P.M.R.B.G. Final results of a prospective randomized trial of bolus meropenem versus infusion meropenem versus ceftazidime/amikacin as empiric initial therapy for infections and fever of unknown origin in neutropenic patients with hematologic malignancies. *Supportive Care in Cancer ;8:Suppl:160. Abstract 64.* **2000**.
24. Hense J, U.M.M.P.H.H.B.G. Prospective randomized trial of bolus meropenem versus infusion meropenem versus ceftazidime/amikacin as empiric initial therapy for infections and fever of unknown origin in neutropenic patients with hematologic malignancies. *Annals of Hematology;77:Suppl 2:S199.* **1998**.

25. Hess, U.; Bohme, C.; Rey, K.; Senn, H.J. Monotherapy with piperacillin/tazobactam versus combination therapy with ceftazidime plus amikacin as an empiric therapy for fever in neutropenic cancer patients. *Support Care Cancer* **1998**, *6*, 402-409, doi:10.1007/s005200050184.
26. Hung, K.C.; Chiu, H.H.; Tseng, Y.C.; Wang, J.H.; Lin, H.C.; Tsai, F.J.; Peng, C.T. Monotherapy with meropenem versus combination therapy with ceftazidime plus amikacin as empirical therapy for neutropenic fever in children with malignancy. *J Microbiol Immunol Infect* **2003**, *36*, 254-259.
27. Jacobs, R.F.; Vats, T.S.; Pappa, K.A.; Chaudhary, S.; Kletzel, M.; Becton, D.L. Ceftazidime versus ceftazidime plus tobramycin in febrile neutropenic children. *Infection* **1993**, *21*, 223-228, doi:10.1007/BF01728894.
28. Jimeno A, A.A.B.S.A.M.L.G.-C.L.C.E.; et al. Randomized study of cefepime versus ceftazidime plus amikacin in patients with solid tumors treated with high dose chemotherapy (HDC) and peripheral blood stem cell support (PBSCS) with febrile neutropenia. *Clinical Transplantation and Oncology ;8 (12):889–95.* **2006**.
29. Jimeno A, A.A.G.C.B.S.C.D.P.-A.L.; et al. Randomized study of cefepime versus ceftazidime plus amikacin in febrile neutropenic patients with solid tumors treated with high dose chemotherapy (HDC) and peripheral blood stem cell support (PBSCS). *Proceedings of the Annual Meeting of the American Society of Clinical Oncology; Abstract 3387.* **2003**.
30. Kamonrattana, R.; Sathitsamitphong, L.; Choeyprasert, W.; Charoenkwan, P.; Natesirinilkul, R.; Fanhchaksai, K. A Randomized, Open-Labeled, Prospective Controlled Study to Assess the Efficacy of Frontline Empirical Intravenous Piperacillin/Tazobactam Monotherapy in Comparison with Ceftazidime Plus Amikacin for Febrile Neutropenia in Pediatric Oncology Patients. *Asian Pac J Cancer Prev* **2019**, *20*, 2733-2737, doi:10.31557/APJCP.2019.20.9.2733.
31. Kiehl Mg, B.M.B.N.G.S.F.A.A. A prospective randomized trial comparing the efficacy and safety of piperacillin/tazobactam versus piperacillin/ tazobactam plus netilmicin in the treatment of tebrile neutropenia in allogeneic stem cell recipients. *Proceedings of the Interscience Conference on Antimicrobial Agents and Chemotherapy.* **2001**.
32. Kinsey, S.E.; Machin, S.J.; Goldstone, A.H. Ceftazidime monotherapy is as effective as ceftazidime combined with gentamicin in the treatment of febrile neutropenic patients. *J Hosp Infect* **1990**, *15 Suppl A*, 49-53, doi:10.1016/0195-6701(90)90079-4.
33. Kliasova G, S.V.L.L.M.L.P.E.T.T.; et al. Monotherapy with meropenem versus combination therapy with ceftazidime plus amikacin as empiric therapy for febrile neutropenic bone marrow transplant patients. *Proceedings of the 11th European Congress of Clinical Microbiology and Infectious Diseases. [MEDLINE: http://www.akm.ch/ eccmid2001/]* **2001**.
34. Kojima, A.; Shinkai, T.; Soejima, Y.; Okamoto, H.; Eguchi, K.; Sasaki, Y.; Tamura, T.; Oshita, F.; Ohe, Y.; Saijo, N. A randomized prospective study of imipenem-cilastatin with or without amikacin as an empirical antibiotic treatment for febrile neutropenic patients. *Am J Clin Oncol* **1994**, *17*, 400-404, doi:10.1097/00000421-199410000-00009.
35. Leyland, M.J.; Bayston, K.F.; Cohen, J.; Warren, R.; Newland, A.C.; Bint, A.J.; Cefai, C.; White, D.G.; Murray, S.A.; Bareford, D.; et al. A comparative study of imipenem versus piperacillin plus gentamicin in the initial management of febrile neutropenic patients with haematological malignancies. *J Antimicrob Chemother* **1992**, *30*, 843-854, doi:10.1093/jac/30.6.843.
36. Lieschke Gj, B.D.R.W.G.M.S.W.M.G.; et al. Imipenem/cilastatin versus tobramycin and piperacillin as initial empiric therapy for febrile episodes in neutropenic patients: interim analysis of a prospective randomized comparison. *Australian and New Zealand Journal of Medicine 1990;3:Suppl 1:424.* **1990**.
37. Liu, C.Y.; Wang, F.D. A comparative study of ceftriaxone plus amikacin, ceftazidime plus amikacin and imipenem/cilastatin in the empiric therapy of febrile granulocytopenic cancer patients. *Chemotherapy* **1989**, *35 Suppl 2*, 16-22, doi:10.1159/000238734.
38. Madasamy, P. 524PD Cefepime versus cefoperazone/sulbactum plus amikacin as empirical antibiotic therapy in cancer patients with febrile neutropenia. *Annals of Oncology* **2016**, *27*, ix170.
39. Pico, J.L.; Marie, J.P.; Chiche, D.; Guiguet, M.; Andremont, A.; Lapierre, V.; Richet, H.; Tancrede, C.; Lagrange, P.; Hayat, M.; et al. Should vancomycin be used empirically in febrile patients with prolonged and profound neutropenia? Results of a randomized trial. *Eur J Med* **1993**, *2*, 275-280.
40. Marie Jp, P.J.L.V.M.C.P.M.C.D.; et al. Comparative trial of ceftazidime alone, ceftazidime + amikacin and ceftazidime + vancomycin as empiric therapy of febrile cancer patients with induced prolonged neutropenia [Traitement empirique des episodes febriles survenant chez les patients cancereux presentant une neutropenie prolongee: essai comparatif ceftazidime seule, ceftazidime+amikacine et ceftazidime+vancomycine]. *Medicine et Maladies Infectieuses ;21:386–8.* **1991**.
41. Marie Jp, P.J.L.C.D.F.F.D.A.B.D.; et al. [Antibiotic therapy protocol using ceftazidime 3g/ day alone or in combination with vancomycin or amikacin. In febrile episodes in neutropenic patients]. [French] [Protocole d'antibiotherie utilisant la ceftazidime a la dose de 3g/jour seule ou en association avec la vancomycin ou l'amikacin]. *Presse Medicale ;17(37):1968–70.* **1988**.
42. Miller, J.A.; Butler, T.; Beveridge, R.A.; Kales, A.N.; Binder, R.A.; Smith, L.J.; Ueno, W.M.; Milkovich, G.; Goldwater, S.; Marion, A.; et al. Efficacy and tolerability of imipenem-cilastatin versus ceftazidime plus tobramycin as empiric therapy of presumed bacterial infection in neutropenic cancer patients. *Clin Ther* **1993**, *15*, 486-499.
43. Morgan, G.; Duerden, B.I.; Lilleyman, J.S. Ceftazidime as a single agent in the management of children with fever and neutropenia. *J Antimicrob Chemother* **1983**, *12 Suppl A*, 347-351, doi:10.1093/jac/12.suppl_a.347.

44. Norrby, S.R.; Vandercam, B.; Louie, T.; Runde, V.; Norberg, B.; Anniko, M.; Andrien, F.; Baudrihaye, M.; Bow, E.; Burman, L.A.; et al. Imipenem/cilastatin versus amikacin plus piperacillin in the treatment of infections in neutropenic patients: a prospective, randomized multi-clinic study. *Scand J Infect Dis Suppl* **1987**, *52*, 65-78.
45. Vandercam, B.; Ezzeddine, H.; Agaliotis, D.; Gala, J.L.; Gigi, J.; Wauters, G.; Michaux, J.L. Imipenem/cilastatin versus piperacillin plus amikacin as empiric therapy in the treatment of febrile episodes in neutropenic patients with haematologic malignancies. *Acta Clin Belg* **1989**, *44*, 99-109, doi:10.1080/17843286.1989.11717996.
46. Novakova, I.; Donnelly, P.; De Pauw, B. Amikacin plus piperacillin versus ceftazidime as initial therapy in granulocytopenic patients with presumed bacteremia. *Scand J Infect Dis* **1990**, *22*, 705-711, doi:10.3109/00365549009027124.
47. Novakova, I.R.; Donnelly, J.P.; de Pauw, B.E. Ceftazidime with or without amikacin for the empiric treatment of localized infections in febrile, granulocytopenic patients. *Ann Hematol* **1991**, *63*, 195-200, doi:10.1007/BF01703442.
48. Ozyilkan, O.; Yalcintas, U.; Baskan, S. Imipenem-cilastatin versus sulbactam-cefoperazone plus amikacin in the initial treatment of febrile neutropenic cancer patients. *Korean J Intern Med* **1999**, *14*, 15-19, doi:10.3904/kjim.1999.14.2.15.
49. Pacheco-Rosas, D.O.; Peregrino-Bejarano, L.; Lopez-Aguilar, J.E.; Juan-Shum, L.; Miranda-Novales, M.G. [Piperacillin/tazobactam plus amikacin vs. piperacilin/tazobactam: treatment for children with febrile neutropenia]. *Rev Med Inst Mex Seguro Soc* **2019**, *57*, 65-73.
50. Papachristodoulou, A.V.M.X.S.P.A.A.C.G. Ceftazidime (CFZ) monotherapy as empirical initial treatment of febrile neutropenia cancer patients (Pts). *Annals of Oncology* **1996**.
51. Perez, C.; Sirham, M.; Labarca, J.; Grebe, G.; Lira, P.; Oliva, J.; Duhalde, M.; Ocqueteau, M.; Acuna, G. [Imipenem/cilastatin versus ceftazidime-amikacin in the treatment of febrile neutropenic patients]. *Rev Med Chil* **1995**, *123*, 312-320.
52. Piccart, M.; Klastersky, J.; Meunier, F.; Lagast, H.; Van Laethem, Y.; Weerts, D. Single-drug versus combination empirical therapy for gram-negative bacillary infections in febrile cancer patients with and without granulocytopenia. *Antimicrob Agents Chemother* **1984**, *26*, 870-875, doi:10.1128/AAC.26.6.870.
53. Ponraj, M.; Dubashi, B.; Harish, B.H.; Kayal, S.; Cyriac, S.L.; Pattnaik, J.; Ranjith, K.; Pillai, U.S.; Jadhav, N.; Matta, K.K.; et al. Cefepime vs. cefoperazone/sulbactam in combination with amikacin as empirical antibiotic therapy in febrile neutropenia. *Support Care Cancer* **2018**, *26*, 3899-3908, doi:10.1007/s00520-018-4260-8.
54. Rolston, K.V.; Berkey, P.; Bodey, G.P.; Anaissie, E.J.; Khardori, N.M.; Joshi, J.H.; Keating, M.J.; Holmes, F.A.; Cabanillas, F.F.; Elting, L. A comparison of imipenem to ceftazidime with or without amikacin as empiric therapy in febrile neutropenic patients. *Arch Intern Med* **1992**, *152*, 283-291.
55. Schuchter L, K.W.P.B.W.J.A.V.D.J.; et al. Ceftazidime vs ticalcillin and gentamicin in febrile neutropenic bone marrow transplant patients: a prospective, randomized, double-blind trial. *Blood 1988; Abstract 1534(Suppl 1):1:406a.* **1988**.
56. Tamura, K.; Matsuoka, H.; Tsukada, J.; Masuda, M.; Ikeda, S.; Matsuishi, E.; Kawano, F.; Izumi, Y.; Uike, N.; Utsunomiya, A.; et al. Cefepime or carbapenem treatment for febrile neutropenia as a single agent is as effective as a combination of 4th-generation cephalosporin + aminoglycosides: comparative study. *Am J Hematol* **2002**, *71*, 248-255, doi:10.1002/ajh.10236.
57. Tamura, K.; Imajo, K.; Akiyama, N.; Suzuki, K.; Urabe, A.; Ohyashiki, K.; Tanimoto, M.; Masaoka, T.; Japan Febrile Neutropenia Study, G. Randomized trial of cefepime monotherapy or cefepime in combination with amikacin as empirical therapy for febrile neutropenia. *Clin Infect Dis* **2004**, *39 Suppl 1*, S15-24, doi:10.1086/383046.
58. Bustamante, C.I. Initial empiric therapy for fever in neutropenia. *Recent Results Cancer Res* **1993**, *132*, 45-56, doi:10.1007/978-3-642-84899-5_4.
59. Jc, W. Antibiotic therapy for the febrile granulocytopenic cancer patient: combination therapy versus monotherapy. *Reviews of Infectious Diseases ;11:Suppl 7:S1572-81.* **1989**.
60. Wade Jc, D.A.F.R.D.G.T.B. Imipenem versus piperacillin plus amikacin, empiric therapy for febrile neutropenic patients: a double blind trial. *Proceedings of the 27th Interscience Conference on Antimicrobial Agents and Chemotherapy. :Abstract No. 1251.* **1987**.
61. Wrzesien-Kus A, J.K.W.A.R.T. Cefepime in monotherapy or in combination with amikacine as the empirical treatment of febrile neutropenic patients. *Acta Haematologica Polonica ;32(2):165–72.* **2001**.
62. Ramphal, R.; Gucalp, R.; Rotstein, C.; Cimino, M.; Oblon, D. Clinical experience with single agent and combination regimens in the management of infection in the febrile neutropenic patient. *Am J Med* **1996**, *100*, 83S-89S, doi:10.1016/s0002-9343(96)00113-1.
63. Yamamura, D.; Gucalp, R.; Carlisle, P.; Cimino, M.; Roberts, J.; Rotstein, C. Open randomized study of cefepime versus piperacillin-gentamicin for treatment of febrile neutropenic cancer patients. *Antimicrob Agents Chemother* **1997**, *41*, 1704-1708, doi:10.1128/AAC.41.8.1704.
64. Yildirim, I.; Aytac, S.; Ceyhan, M.; Cetin, M.; Tuncer, M.; Cengiz, A.B.; Secmeer, G.; Yetgin, S. Piperacillin/tazobactam plus amikacin versus carbapenem monotherapy as empirical treatment of febrile neutropenia in childhood hematological malignancies. *Pediatr Hematol Oncol* **2008**, *25*, 291-299, doi:10.1080/08880010802016847.
65. Zengin, E.; Sarper, N.; Kilic, S.C. Piperacillin/tazobactam monotherapy versus piperacillin/tazobactam plus amikacin as initial empirical therapy for febrile neutropenia in children with acute leukemia. *Pediatr Hematol Oncol* **2011**, *28*, 311-320, doi:10.3109/08880018.2011.557144.
